# Pollinator efficiency, rather than bee decline, explains a shift to hummingbird pollination in tropical montane forests

**DOI:** 10.1111/nph.71291

**Published:** 2026-06-04

**Authors:** Pedro Juárez, Kathryn Gerhardt, Eden Hughes, Cecilia Girvin, Anise Dellith‐Moser, Dennis Tenorio, Annya Livak, Kathleen M. Kay

**Affiliations:** ^1^ Department of Ecology and Evolutionary Biology University of California Santa Cruz CA 95060 USA; ^2^ Department of Biology Lund University 223 62 Lund Sweden; ^3^ Department of Biological Sciences California State Polytechnic University Humboldt Arcata CA 95521 USA; ^4^ Smithsonian Tropical Research Institute Balboa Ancón Panama Republic of Panama

**Keywords:** *Costus*, euglossine bee, floral evolution, hummingbird, pollen‐transfer efficiency, pollination shift, pollination syndromes, speciation

## Abstract

- A longstanding but untested hypothesis proposes that reduced bee visitation in tropical montane cloud forests has repeatedly driven the evolution of hummingbird pollination. Here, we test whether recently diverged bee and hummingbird pollination syndromes in two sister species are adapted to their pollination environments, and whether this reflects declining bee activity at higher elevations. Alternatively, we ask whether higher pollen transfer efficiency drives adaptation to hummingbirds regardless of bee availability.
- We measured visitation and per‐visit efficiency to estimate pollinator effectiveness and conducted reciprocal translocations of *Costus kuntzei*, with ancestral bee pollination, and *Costus wilsonii*, with derived hummingbird pollination, across an elevational gradient in Costa Rica, including sites within and outside each species' range and at their elevational boundary.
- In their ranges, the species are specialized on bees or hummingbirds. However, pollinator effectiveness was higher for hummingbird‐pollinated *C. wilsonii* because of greater per‐visit efficiency, despite bee‐pollinated *C. kuntzei* experiencing higher visitation. In reciprocal translocations, *C. kuntzei* showed uniform bee visitation across habitats, whereas hummingbird visitation increased with elevation for *C. wilsonii*.
- Our results show that floral adaptation to hummingbird pollination is likely driven by higher hummingbird visitation in montane environments combined with greater per‐visit efficiency, rather than declining bee visitation with elevation.

## Graphical abstract

Hummingbird pollination is a hallmark of American plant diversity and has long been thought to evolve in tropical mountains due to declining bee activity. Using sister species of *Costus* specialized on bees (*C. kuntzei*) and hummingbirds (*C. wilsonii*), we show that this shift is not driven by reduced bee visitation with elevation, but by greater pollination effectiveness of hummingbirds. Photographs by P. Juárez.

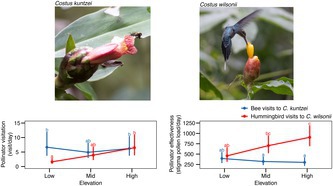

## Resumen

- Una hipótesis antigua, pero aún no evaluada, propone que la reducción en las visitas de abejas en los bosques nubosos tropicales de montaña ha impulsado repetidamente la evolución de la polinización por colibríes. Aquí evaluamos si los síndromes de polinización por abejas y por colibríes, recientemente divergentes en dos especies hermanas, están adaptados a sus ambientes de polinización, y si esto refleja una disminución en la actividad de las abejas a mayores elevaciones. Alternativamente, evaluamos si una mayor eficiencia en la transferencia de polen impulsa la adaptación a los colibríes independientemente de la disponibilidad de abejas.
- Medimos la visitación y la eficiencia por visita para estimar la efectividad de los polinizadores, y realizamos translocaciones recíprocas de *Costus kuntzei*, con polinización ancestral por abejas, y *Costus wilsonii*, con polinización derivada por colibríes, a lo largo de un gradiente elevacional en Costa Rica, incluyendo sitios dentro y fuera del rango de distribución de cada especie y en su límite elevacional.
- Dentro de sus rangos de distribución, las especies están especializadas en abejas o colibríes. Sin embargo, la efectividad de los polinizadores fue mayor para *C. wilsonii*, polinizada por colibríes, debido a una mayor eficiencia por visita, a pesar de que *C. kuntzei*, polinizada por abejas, recibió una mayor visitación. En las translocaciones recíprocas, *C. kuntzei* mostró una visitación uniforme por abejas entre hábitats, mientras que la visitación por colibríes aumentó con la elevación en *C. wilsonii*.
- Nuestros resultados muestran que la adaptación floral a la polinización por colibríes probablemente está impulsada por una mayor visitación de colibríes en ambientes montanos combinada con una mayor eficiencia por visita, más que por una disminución en la visitación de abejas con la elevación.

## Introduction

Pollinator‐driven adaptation is predicted to drive floral divergence, contributing to floral isolation and hence speciation (Wilson & Thomson, 1996; Waser & Campbell, 2004; Johnson, 2006, 2025; Sun *et al*., 2014; Newman *et al*., 2015; Gross *et al*., 2023). Such floral adaptation is particularly important in pollination shifts, wherein suites of floral traits associated with specific pollinator functional groups (i.e. pollination syndromes) adapt to new pollinators, allowing plants to exploit alternative pollination niches (Fenster *et al*., 2004; Kay & Sargent, 2009; Van der Niet *et al*., 2014; Phillips *et al*., 2020; Johnson, 2025). Pollination shifts may be an important mechanism driving plant speciation because they often involve qualitative changes in floral traits that cause substantial reproductive isolation (Johnson, 2006, 2025; Kay & Sargent, 2009; Van der Niet *et al*., 2014). However, understanding how adaptive processes generate pollination shifts remains challenging because these shifts are typically studied retrospectively, after divergence (Grant & Grant, 1965; Kay & Anderson, 2025).

The hummingbird pollination syndrome has evolved many times in different plant lineages and habitats across the Americas, primarily from bee‐pollinated ancestors (Dellinger *et al*., 2023; Barreto *et al*., 2024). It is associated with adaptation to specific habitats, although the particular habitats differ between temperate and tropical biomes (Grant & Grant, 1968; Stebbins, 1990; Dellinger *et al*., 2023). The Neotropics harbor the highest diversity of hummingbird‐adapted plants, most of which occur in montane cloud forests and páramo habitats, typically above 1500 m asl, where moist conditions and high cloud cover prevail (Stiles, 1981; Dellinger *et al*., 2023). The repeated evolution of hummingbird pollination in these environments is thought to reflect floral adaptation toward more effective pollinators in high‐elevation habitats, where it has been hypothesized that bees are less available than at low elevations (Cruden, 1972; Dellinger *et al*., 2021, 2023). Limited bee availability, reflected in lower visitation rates, is thought to result from environmental constraints, such as low temperatures caused by elevation and cloud cover, that restrict bee foraging activity (Inouye, 1975; Armbruster & McCormick, 1990; Armbruster & Berg, 1994). By contrast, hummingbirds are considered more reliable pollinators because they are less sensitive to those conditions (Cruden, 1972; Dellinger *et al*., 2021). Nevertheless, some tropical bees are capable of endothermic warming (May & Casey, 1983; Stone, 1993; Armbruster & Berg, 1994), cold‐adapted bees are known to behaviorally regulate their body temperature (Heinrich & Vogt, 1993), and there is little empirical evidence that bee activity is, in fact, constrained in montane cloud forests.

Alternatively, shifts to hummingbird pollination may be driven by greater hummingbird pollen transfer efficiency rather than limited bee visitation in certain habitats (Thomson & Wilson, 2008; Ohashi *et al*., 2021). Under this hypothesis, bees may still be frequent visitors, yet hummingbird pollination may represent a higher adaptive peak if hummingbirds consistently transfer pollen more efficiently per visit (Aigner, 2001; Kay & Anderson, 2025). Thus, unlike the limited bee availability hypothesis (Cruden, 1972), this hypothesis predicts that floral adaptation is driven by differences in pollinator effectiveness rather than by differences in visitation frequency alone. In this case, the most frequent or abundant pollinators do not necessarily contribute the most to reproductive success (Fenster *et al*., 2004). Consequently, adaptation to hummingbird pollination in montane environments may not be driven by bee visitation *per se*, but by pollinators that transfer pollen more efficiently in those habitats (Kay & Anderson, 2025).

**Fig. 1:**
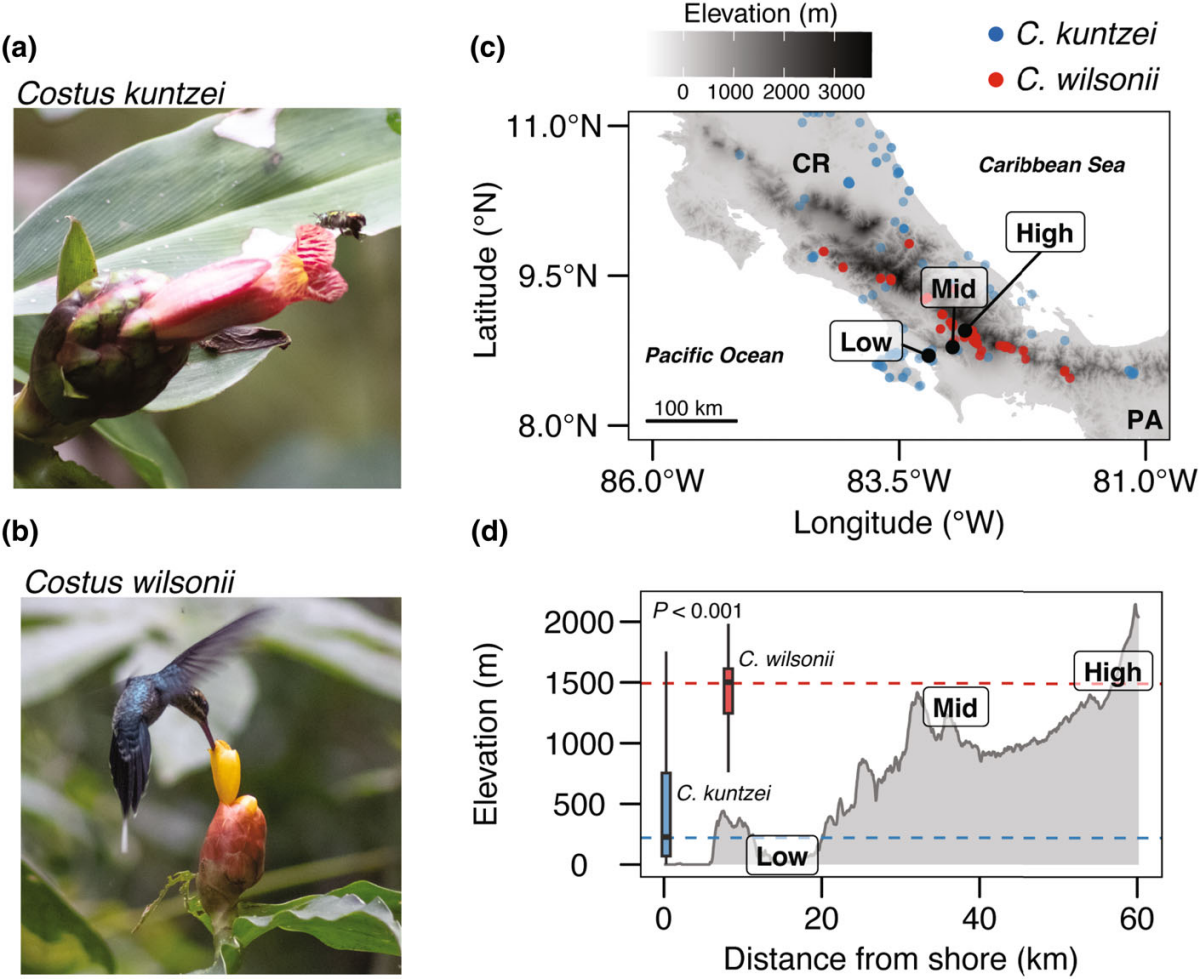
Species information and study sites are shown. (a) *Costus kuntzei* and a male euglossine bee (*Euglossa flammea*) hovering before visiting. (b) *Costus wilsonii* being visited by a female green hermit (*Phaethornis guy*). (c) Elevation map of Costa Rica (CR) and western Panama (PA) showing our study sites and occurrence points based on herbarium specimen data. (d) Elevational gradient profile showing the distribution of study sites across elevations. Colored dashed lines represent the median elevation of each species. Boxplots show elevation ranges derived from occurrences of *C. kuntzei* (median elevation = 221 m, median absolute deviation = ±245 m, N specimens = 204) and *C. wilsonii* (median elevation = 1493 m, median absolute deviation = ±255 m, N specimens = 63; W = 8802, *P*‐value <0.001). We performed a Wilcoxon rank‐sum test to compare median elevation between species. Photographs (a, b) by P. Juárez.

In this study, we investigate the mechanisms driving adaptive floral divergence in two sister species of Neotropical spiral gingers with a recent pollination shift from bee to hummingbird pollination. *Costus kuntzei* inhabits lowland tropical rainforest and exhibits a euglossine bee pollination syndrome, which is ancestral for this clade of plants, and *Costus wilsonii* inhabits montane cloud forest and exhibits a derived hermit hummingbird pollination syndrome (Vargas *et al*., 2020; Kay & Grossenbacher, 2022; Fig. 1). We test whether each species is adapted to its home pollination environment and whether that adaptation is due to differences in visitation rates, pollen transfer efficiency, or both. First, we assess pollinator visitation rates and per‐visit pollen deposition efficiency for naturally occurring individuals of each plant species within their respective geographic ranges. Specifically, we quantify both pollinator visitation (visits per day to their single‐day flowers) and per‐visit efficiency (stigma pollen load per visit) to then estimate overall pollinator effectiveness (stigma pollen load per day) as their product (Armbruster *et al*., 2000; Fenster *et al*., 2004). We predict that visitation might be higher for the bee‐pollinated species, but that greater hummingbird per‐visit efficiency would result in comparable or higher total effectiveness for the hummingbird‐pollinated species.

Second, we conduct a reciprocal translocation experiment to directly test whether pollination systems are adapted to their home pollination environments, and whether that adaptation is driven by declining bee activity at higher elevations, differences in pollen transfer efficiency between bees and hummingbirds, or both. To accomplish this, we translocate standardized floral arrays across sites within and outside the range of each species and at their elevational range boundary. We then compare pollinator visitation rates and total effectiveness between and within species across elevations. According to the hypothesis that bees are less available at higher elevations, bee visitation rates should decline with elevation (Cruden, 1972). Alternatively, accounting for per‐visit efficiency in addition to visitation rates may reveal that pollination systems are adapted to their home pollination environments, even in the absence of substantial differences in bee visitation.

## Materials and Methods

### Study species and natural history

*Costus kuntzei* K. Schum. and *Costus wilsonii* Maas (Costaceae) are sister species of large, perennial understory monocots that differ in elevation, habitat, and pollination syndrome (Fig. 1). *Costus wilsonii* represents one of the most recent shifts from bee to hummingbird pollination in the Neotropical *Costus*; which exhibits at least 10 independent transitions from bee to hummingbird pollination (Kay *et al*., 2005; Vargas *et al*., 2020; Kay & Grossenbacher, 2022; Valderrama *et al*., 2022). *Costus kuntzei* is visited primarily by euglossine bees, mainly long‐tongued species in the genera *Euglossa* and *Eulaema* (tribe Euglossini), whereas *C. wilsonii* is specialized on the green hermit hummingbird (*Phaethornis guy*, subfamily Phaethornithinae; Supporting Information Video S1).

*Costus kuntzei* exhibits the ancestral bee pollination syndrome. It has large, pale white‐to‐pink flowers with a broad, expanded labellum that serves as a landing platform and exhibits yellow nectar guides and conspicuous red striations (Maas, 1972; Kay & Grossenbacher, 2022). *Costus kuntzei* flowers produce volatiles that attract euglossine bees (Darragh *et al*., 2025). This species is widespread, inhabiting lowland tropical rainforests of Central America and northwestern South America, from sea level to mid elevations (Fig. 1; Methods S1).

*Costus wilsonii* has shifted to a hermit hummingbird pollination syndrome. It has unscented, smaller, curved, yellow flowers with reduced labella and reduced or absent red striations (Maas, 1977; Kay & Grossenbacher, 2022; Darragh *et al*., 2025). *Costus wilsonii* is endemic to a region of the montane cloud forests of the Talamanca Cordillera and La Fila Brunqueña in southern Central America, ranging from central Costa Rica to northwestern Panama, at mid‐to‐high elevations (Fig. 1; Methods S1).

The inflorescences of both species usually produce a single flower per day, which opens near dawn and senesces in the afternoon (Schemske, 1981; Stratton, 1989). The typical lifespan of an individual *Costus* flower is from *c*. 05:00 to 14:00 h (Stratton, 1989; Armbruster & McCormick, 1990; Stiles, 1995; Kay & Schemske, 2003). Flowers are adichogamous (Ramirez & Seres, 1994), meaning that anthers dehisce and the stigma becomes receptive at the same time. *Costus kuntzei* flowers are significantly larger than those of *C. wilsonii* for most traits, including having larger ovaries with more ovules and larger anthers with presumably more pollen. Nectar volume, however, is significantly lower in *C. kuntzei*, whereas tube diameter and sugar concentration do not differ between species. We further characterize the floral traits of both species in Table S1.

### Study sites

We selected three study sites across an elevational gradient in Puntarenas province in southern Costa Rica, including sites within and outside the range of each species, and at their elevational range boundary (Fig. 1). The low‐elevation site is located in a lowland tropical rainforest, where only *C. kuntzei* occurs, at La Gamba Field Station, Golfo Dulce region (8°42′03.78″ N, 83°12′06.14″ W 70 m asl), adjoined to Piedras Blancas National Park at the Pacific southern coast of Costa Rica. The climate exhibits high precipitation (5900 mm yr^−1^) and high temperatures (*c*. 28°C) (Zamora, 2001). The mid elevation site is located in a transitional premontane forest where *C. kuntzei* and *C. wilsonii* naturally co‐occur in a narrow elevational band, located at Las Cruces Biological Station and Botanical Garden (08°47′03.52″ N, ‐82°57′34.00″ W; 1300 m asl), along Fila Cruces, a spur of La Fila Brunqueña near the pacific southern coast of Costa Rica. The climate exhibits relatively moderate precipitation (3600 mm yr^−1^) and temperatures (*c*. 22°C) (Janzen, 1983). The high‐elevation site is located in a montane cloud forest where only *C. wilsonii* occurs, at Finca Las Alturas del Bosque Verde (08°57′05.07″ N, ‐082°44′34.22″ W; 1600 m asl), in the southern sector of the Talamanca Cordillera in Costa Rica, adjoined to Las Tablas Protected Zone and La Amistad International Park near the border to Panama. The climate exhibits lower and highly seasonal precipitation (2000–3000 mm yr^−1^) and lower temperatures (*c*. 19°C) (Herrera, 2016). Because we sampled only one site at each elevation, elevation and site are confounded in our design. However, we sampled many widely spaced naturally occurring plants at each site and established many separate locations for arrays within each site in order to capture within‐site variation in pollinator interactions.

### How effective are hummingbirds and bees in natural populations of each plant species?

We studied pollinator effectiveness during the peak of the flowering season, from June to July 2018–2024, at three study sites. We define pollinator effectiveness (PE) as the product of visitation rate (V) and per‐visit pollen transfer efficiency (E) (Armbruster *et al*., 2000; Fenster *et al*., 2004). Pollinator visitation was quantified as the number of legitimate visits to individual plants in the field, filmed during the hours spanning 05:00 and 14:00 h, which is the main period of pollinator activity and the typical lifespan of an individual *Costus* flower (Stratton, 1989; Armbruster & McCormick, 1990; Stiles, 1995; Kay & Schemske, 2003). Because *Costus* can propagate vegetatively (Edeoga & Okoli, 1996), conspecifics were sampled from distances ranging from tens of meters to several kilometers to avoid sampling the same genets.

We used Canon PowerShot SX420 IS and SX530 HS digital cameras with auto‐controlled aperture settings. To automate motion detection, we used the Canon Hack Development Kit (CHDK, https://chdk.fandom.com) with the MDFB2013 motion detection script, setting the trigger threshold to 2–5 and enabling burst modes of up to 30 s. A total of 931.38 observation hours from 143 flowers were filmed (mean observation time per flower per day ± SD = 6.52 ± 1.92 h). This includes a total of 458.78 h from 67 flowers of *C. kuntzei*, with 230.08 h from 33 flowers from low elevation and 228.70 h from 34 flowers from mid elevation; and a total of 476.91 h from 76 flowers of *C. wilsonii*, with 199.99 h from 34 flowers from mid elevation and 276.92 h from 42 flowers from high elevation. See Supplemental Information, Tables S2 and S3, for details of pollinator visitation sampling. We only considered legitimate pollinators who reliably contacted both the anthers and stigma. Specifically, we classified bees as pollinators when they crawled in and out of the floral gullet of *C. kuntzei* and hummingbirds as pollinators when they inserted and retracted their beaks from *C. wilsonii* flowers. Given the morphology of these flowers, it can reliably be presumed that contact would be made with the anthers and stigma on such visits, and in many videos, we could see visible pollen on the animal at the end of a visit. Video footage was analyzed using VLC media player (v.3.0.20, https://www.videolan.org/vlc/).

Pollinator per‐visit efficiency (*E* = *L*/*V*_n_) was quantified as the total number of conspecific pollen grains deposited on individual stigmas, or stigma pollen load (*L*), divided by the number of legitimate visits recorded during a period of observation (*V*_n_), with visits assigned to pollinator functional groups (bees for *C. kuntzei* and hummingbirds for *C. wilsonii*), representing the stigma pollen load per visit. Grouping visits by pollinator functional group is justified because pollination syndromes are based on the hypothesis that pollinators can be classified into functional categories whose members behave similarly on flowers and therefore exert similar selection on floral traits (Fenster *et al*., 2004). Floral visitors were filmed using the methods outlined above between 05:00 and 11:00 h for each species in June of 2024. Of all the flowers used to measure visitation rates, 77.94% were also used to measure pollinator per‐visit efficiency. We supplemented sample sizes by allowing pollinators to visit potted plants placed in forest gaps and edges. We ensured a minimum distance of *c*. 25 m from the nearest naturally occurring plant or potted individual. Four flowers with no visits were discarded from our analysis of pollinator efficiency. Those flowers had an average of four pollen grains on their stigmas, showing that pollen receipt is dependent on visitation. For this efficiency data, we filmed a total of 566 observation hours, including 244 h from 27 individuals of *C. kuntzei* and 322 h from 24 individuals of *C. wilsonii* (mean observation time per individual flower per day ± SD = 6.08 ± 1.20 h). After each observation period, stigmas were collected individually in 1.5 ml microcentrifuge tubes containing 1 M NaOH and softened for 8 h. Stigmas were then mounted on microscope slides using basic‐fuchsin‐tinted glycerin jelly to quantify pollen loads (number of conspecific pollen grains) under a microscope (Beattie, 1971). Conspecific pollen was identified using prepared reference samples of focal species. Additionally, we prepared reference samples of a few other species in the area known to be visited by the same or similar pollinators. We also verified there were no other co‐flowering *Costus* species in the area sharing a pollination syndrome, as pollen can be indistinguishable among congeners (Punt, 1968). A total of 97 stigmas were analyzed, including 17 stigmas of *C. kuntzei* from low elevation, 26 stigmas of *C. kuntzei* and 18 stigmas of *C. wilsonii* from mid elevation, and 36 stigmas of *C. wilsonii* from high elevation. See Table S4, for details of pollinator efficiency sampling.

We quantified pollinator effectiveness (PE = V × E), or per‐day stigmatic pollen load, as the product of per‐day visitation (V) and per‐visit efficiency (E) (Armbruster *et al*., 2000; Fenster *et al*., 2004) of the corresponding pollinator functional group on each plant species (bees for *C. kuntzei* and hummingbirds for *C. wilsonii*), representing a measure of pollinator performance. This approach was used instead of directly measuring total per‐day stigmatic pollen load because visitation was recorded on more flowers and across more days, providing a more representative estimate of pollinator visitation rates. Pollinator efficiency can be variable; hence, we implemented bootstrapping to incorporate variation in pollinator efficiency when estimating effectiveness (Manly, 2018). To ensure species‐specific estimates, we first filtered both pollinator visitation rate and per‐visit efficiency datasets by plant species (*C. kuntzei* and *C. wilsonii*). For each species, we extracted the observed distribution of pollinator per‐visit efficiency values and resampled values with replacement. Specifically, for each visitation observation, we generated 10 000 bootstrap replicates of efficiency values by randomly resampling from the observed species‐specific efficiency dataset. Each resampled efficiency value was then multiplied by the corresponding per‐day visitation rate in the visitation dataset, producing a distribution of possible PE values for each flower per observation day. The final effectiveness estimate for each observation was calculated as the mean of the bootstrapped values.

### Are plant species adapted to their native pollination environment, and is this driven by declining bee visitation with elevation?

We examined whether pollination systems are adapted to their native pollination environments by conducting a reciprocal translocation experiment between *C. kuntzei* and *C. wilsonii*, exposing floral arrays to quantify pollinator visitation and effectiveness across an elevational gradient (Fig. 1). Rhizomes of *C. kuntzei* and *C. wilsonii* were collected from their native populations and grown to flowering in fabric shadehouses at the low‐ and mid‐elevation sites in pots ranging from *c*. 11.4 l to 26.5 l. Plants were transported by truck to the high‐elevation site, where constructing a shadehouse was not feasible. To control for intraspecific floral variation within the arrays and to standardize floral composition across sites, each array included one *C. kuntzei* plant from low elevation, one *C. kuntzei* plant from mid elevation, one *C. wilsonii* plant from mid elevation, and one *C. wilsonii* plant from high elevation. To avoid contaminating the local gene pool, all experimental plants were emasculated daily before exposure. Emasculation does not affect pollinator preference since it is not visible from outside the flower, and all pollinators are known to feed on nectar (although some female euglossine bees also collect pollen by grooming after visits).

Pollinators were filmed between 05:00 and 14:00 h using the filming methods outlined above for each floral array at all study sites in June and July 2021–2023. Floral arrays were placed in forest gaps and edges, ensuring a minimum distance of *c*. 50–100 m from the nearest array. A total of 900.46 observation hours were filmed at experimental arrays, including 263.98 h from seven arrays at low elevation, 319.83 h from seven arrays at mid elevation, and 316.65 h from seven arrays at high elevation (overall mean observation time per array‐day ± SD = 8.83 ± 0.96 h). Pollinator visitation (V) and effectiveness (PE) were estimated using the same methods outlined above, with PE calculated using bootstrapped values from the pollinator per‐visit efficiency (E) dataset. See Tables S5 and S6, for details of reciprocal translocation experiment sampling.

### Statistical analysis

Statistical analyses were conducted in the R statistical environment v.4.4.1 (R Core Team, 2021). We used generalized linear mixed‐effects models (GLMM) throughout, implemented with *glmmTMB* (Brooks *et al*., 2017). Across all models, we computed estimated marginal means to assess significance in pairwise comparisons, adjusting *P*‐values with a Sidak *post‐hoc* test for multiple comparisons using *emmeans* (Lenth, 2022). Model fits were evaluated using *DHARMa* (Hartig, 2022) and compared using multimodel inference based on the Akaike Information Criterion (AIC) with *AICcmodavg* (Mazerolle & Mazerolle, 2017).

To compare components of PE between species, we first fitted a GLMM using a negative binomial distribution to assess pollinator visitation (visits per day) differences between *C. kuntzei* and *C. wilsonii*. The model included the number of visits per day as the response variable, plant species (*C. kuntzei* and *C. wilsonii*) as the predictor, plant individual and year as random effects to account for annual variation in visitation, and the logarithm of observation hours for that day as an offset, since the exact duration of daily observation varied slightly. We did not include a zero‐inflated term because visits were generally rare but did not show an excess of zeros, and many observation periods consisted of single visits. This pattern may be consistent with traplining pollinator visitation being infrequent yet regular (Gill, 1988). Next, we compared pollinator per‐visit efficiency on *C. kuntzei* and *C. wilsonii* stigmas by fitting a GLMM with a lognormal distribution. In this model, pollinator per‐visit efficiency (stigma pollen load per visit) was the response variable, plant species (*C. kuntzei* and *C. wilsonii*) was the predictor, individual was a random effect, and the logarithm of observation hours was included as an offset. We did not include year as a random effect because all measurements of pollinator per‐visit efficiency were made in June 2024. We then fitted a GLMM with a lognormal distribution to assess differences in PE (stigma pollen load per day) between *C. kuntzei* and *C. wilsonii*. This model included PE estimates as the response variable, plant species (*C. kuntzei* and *C. wilsonii*) as the predictor, plant individual as a random effect, and the logarithm of observation hours as an offset. Additionally, to compare components of PE between species across natural populations, we fitted the same GLMMs outlined above but using plant species (*C. kuntzei* and *C. wilsonii*), origin site (low, mid, high), and their interaction (plant species × site) as predictors.

To assess pollinator visitation in the translocation experiment, we fitted a GLMM with a zero‐inflated negative binomial distribution including the number of visits as the response variable, plant species (*C. kuntzei* and *C. wilsonii*), site (low, mid, high), and their interaction (plant species × site) as predictors, floral array number and year as random effects to account for spatial position and annual variation in visitation, and the logarithm of observation hours as an offset. We then fitted a GLMM with a lognormal distribution to analyze PE in the translocations. This model included PE estimates as the response variable, plant species (*C. kuntzei* and *C. wilsonii*), site (low, mid, high), and their interaction (plant species × site) as predictors, floral array number as a random effect, and the logarithm of observation hours as an offset.

## Results

### Hummingbird‐adapted plants receive more pollen despite lower visitation rates

For naturally occurring individuals, *C. kuntzei* was visited by euglossine bees, while *C. wilsonii* was mainly visited by hermit hummingbirds. No hummingbird visits to *C. kuntzei* or bee visits to *C. wilsonii* were recorded. *Costus kuntzei* pollinators at low elevation included two *Euglossa* species (*Eug. imperialis* and *Eug. flammea*), *Eulaema polychroma*, and a single visit by *Exaerete frontalis*. At mid elevation, *C. kuntzei* pollinators included two *Euglossa* species (*Eug. asarophora* and *Eug. flammea*) and three *Eulaema* species (*Eul. polychroma*, *Eul. bombiformis*, and *Eul. cingulata*). *Costus wilsonii* pollinators at mid elevation included *Phaethornis guy* (subfamily Phaethornithinae), with a single visit from *Campylopterus hemileucurus* (subfamily Trochilinae). At high elevation, *C. wilsonii* was visited exclusively by *P. guy*.

**Fig. 2:**
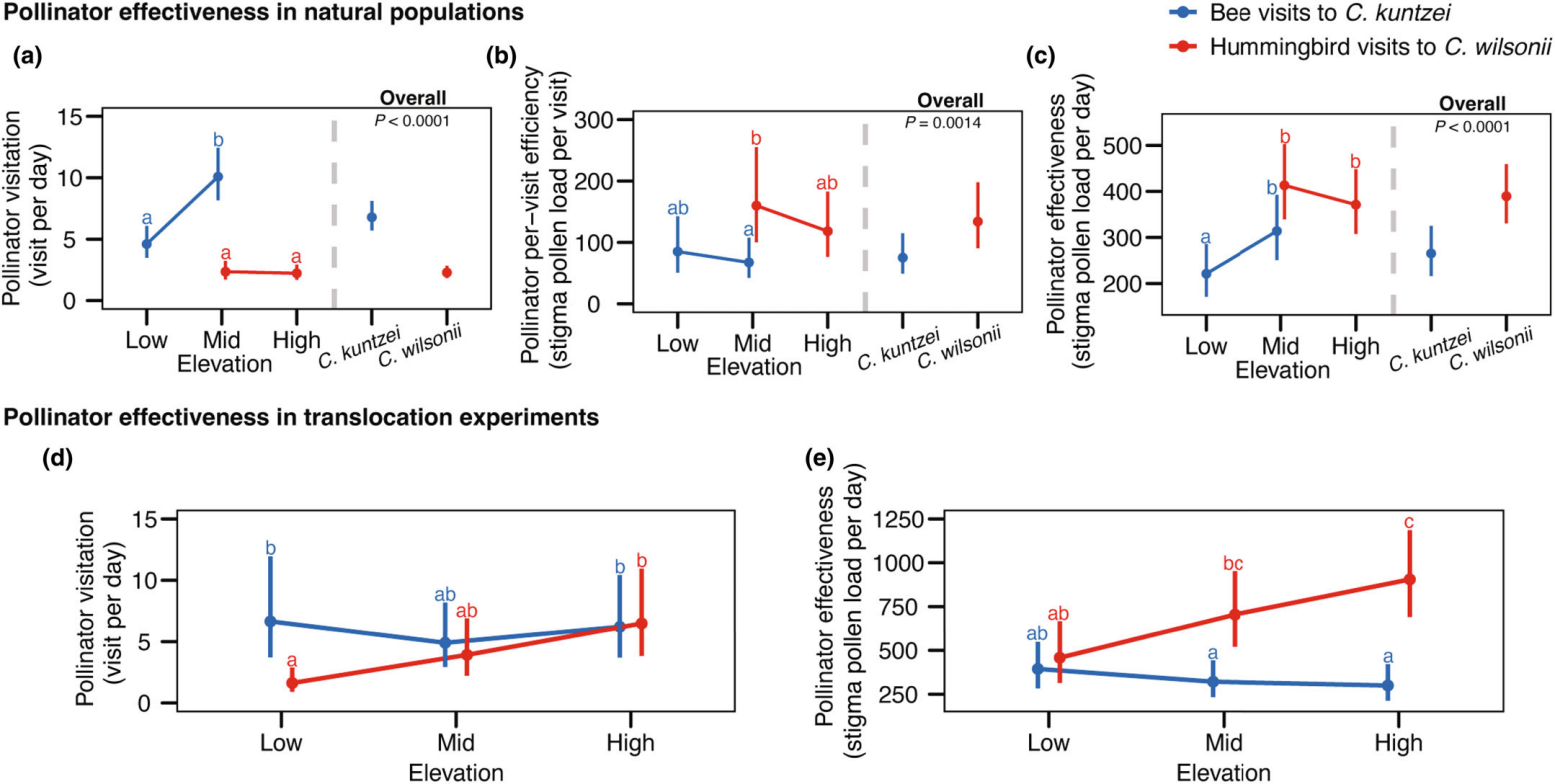
Measurements from natural populations and reciprocal translocations indicate that the shift to hummingbird pollination is not driven by declining bee visitation, but by greater hummingbird effectiveness. (a) Pollinator visitation rates (visits per day) in natural populations showing significantly higher bee visitation for *Costus kuntzei*. (b) Pollinator per‐visit efficiency (stigma pollen load per visit) indicating greater per‐visit efficiency by hummingbirds for *C. wilsonii*. (c) Overall pollinator effectiveness (stigma pollen load per day) showing higher hummingbird effectiveness for *C. wilsonii*. (d) Pollinator visitation to translocated arrays across elevations, showing higher bee visitation for *C. kuntzei* at low elevation but no significant differences at mid‐ and high elevations. (e) Pollinator effectiveness across elevations, indicating no difference at low elevation but greater hummingbird effectiveness for *C. wilsonii* at mid‐ and high elevations. All means were estimated from generalized linear models (GLMM). Means not sharing any letter are significantly different at the *P* < 0.05 level of significance, adjusted by Sidak *post‐hoc* test for multiple comparisons. Error bars indicate 95% confidence intervals.

*Costus kuntzei* and *C. wilsonii* differ in their overall pollinator visitation rate (visits per day), pollinator per‐visit efficiency (stigma pollen load per visit), and PE (stigma pollen load per day; Fig. 2). On average, *C. kuntzei* received significantly more bee visits per day (mean = 6.80 visits per day, 95% CI: 5.50–8.41) than hummingbird visits on *C. wilsonii* (mean = 1.98 visits per day, 95% CI: 1.56–2.52; *z* = 7.501, *P* < 0.0001; Fig. 2a; Table S7). By contrast, *C. wilsonii* received significantly more pollen grains per hummingbird visit (mean = 134.0 pollen grains per visit, 95% CI: 90.7–198) than *C. kuntzei* received per bee visit (mean = 75.2 pollen grains per visit, 95% CI: 49.1–115; *z* = −3.193, *P* = 0.0014; Fig. 2b; Table S8). Similarly, we found significantly higher PE for hummingbirds on *C. wilsonii* (mean = 389 pollen grains per day, 95% CI: 330–458) compared to bees on *C. kuntzei* (mean = 265 pollen grains per day, 95% CI: 216–325; *z* = −4.398, *P* < 0.0001; Fig. 2c; Table S9).

Contradicting the prediction that bee availability decreases as elevation increases (Cruden, 1972), *C. kuntzei* received significantly more bee visits per day at mid elevation (mean = 10.07, 95% CI: 7.82–12.98) compared to low elevation (mean = 3.45, 95% CI: 2.57–4.63; *z* = −5.402, *P* < 0.0001; Fig. 2a; Table S10). *Costus kuntzei* received similar bee per‐visit efficiency at mid elevation (mean = 67.4, 95% CI: 37.2–122) compared to low elevation (mean = 85.1, 95% CI: 44.1–164; z = 0.872, *P* = 0.8194; Fig. 2b; Table S11). Consistent with higher bee visitation rates at mid elevation, *C. kuntzei* received significantly higher bee effectiveness (stigma pollen load per day) at mid elevation (mean = 314, 95% CI: 251–392) compared to low elevation (mean = 220, 95% CI: 171–285; z = −2.573, *P* = 0.0494; Fig. 2c; Table S12).

Hummingbird visitation rates to *C. wilsonii* did not significantly differ between mid‐ (mean = 1.94, 95% CI: 1.39–2.72) and high elevation (mean = 2.01, 95% CI: 1.50–2.71; *z* = −0.158, *P* = 0.9986; Fig. 2a; Table S10). *Costus wilsonii* received similar hummingbird per‐visit efficiency at mid and high elevation (high mean = 118.2, 95% CI: 68.0–206; mid mean = 160, 95% CI: 88.4–290; z = −1.245, *P* = 0.5972; Fig. 2b; Table S11). Therefore, because of similar visitation rates and per‐visit efficiency between populations, hummingbird effectiveness for *C. wilsonii* did not differ between mid‐ (mean = 413, 95% CI: 340–503) and high elevation (mean = 370, 95% CI: 306–446; *z* = 1.038, *P* = 0.7270; Fig. 2c; Table S12).

### Visitation and pollinator effectiveness of hummingbird‐adapted plants increase with elevation, whereas both remain constant in bee‐adapted plants

Pollinator identity in the translocated arrays was similar to that observed under natural conditions. *Costus kuntzei* pollinators at high elevation included *Eug. flammea* and *Eul. polychroma*, which were common visitors within its home range. *Costus wilsonii* at low elevation was visited exclusively by *Phaethornis longirostris*, a hermit hummingbird species common in lowlands but rare above mid elevations (Garrigues & Dean, 2014). *Phaethornis longirostris* is functionally and ecologically similar to *P. guy*, which was *C. wilsonii*'s primary visitor within its home range. Both are large forest understory hermit hummingbirds with similar behavior and morphology (Skutch, 1964; Stiles & Skutch, 1989).

Similar to what was seen in the observational study, and thus, also contrary to the prediction of the bee availability hypothesis (Cruden, 1972), bee visitation rates (visits per day) to *C. kuntzei* did not differ significantly across elevations (low elevation mean = 6.65, 95% CI: 3.70–12.0; mid elevation mean = 4.90, 95% CI: 2.93–8.20; *z* = 0.767, *P* = 0.9731; low elevation vs high elevation mean = 6.21, 95% CI: 3.70–10.4; *z* = 0.173, *P* = 1). By contrast, hummingbird visitation rates for *C. wilsonii* significantly increased with elevation, from low elevation (mean = 1.63, 95% CI: 0.91–2.90) to high elevation (mean = 6.48, 95% CI: 3.84–11.0; *z* = −3.473, *P* = 0.0068), although the increase from low‐ to mid‐elevation (mean = 3.92, 95% CI: 2.23–6.90) was not statistically significant (*z* = −2.127, *P* = 0.2730). Between species across elevations, *C. kuntzei* and *C. wilsonii* differed in their pollinator visitation rate at low elevation but not at mid and high elevations (Fig. 2d; Table S13). At low elevation, we found a higher bee visit rate for *C. kuntzei* (mean = 6.65, 95% CI: 3.70–12.00) compared to the hummingbird visit rate for *C. wilsonii* (mean = 1.63, 95% CI: 0.91–2.90; *z* = 6.267, *P* < 0.0001). By contrast, we did not find significant differences in pollinator visit rates between species at mid‐ (*C. kuntzei* mean = 4.90, 95% CI: 2.93–8.20; *C. wilsonii* mean = 3.92, 95% CI: 2.23–6.90; *z* = 0.974, *P* = 0.9264) and high elevations (*C. kuntzei* mean = 6.21, 95% CI: 3.69–10.40; *C. wilsonii* mean = 6.48, 95% CI: 3.83–11.00; *z* = −0.203, *P* = 1).

We compared overall PE across translocation sites by adjusting pollinator visitation rates with per‐visit efficiency (Fig. 2e; Table S14). At low elevation, we found no significant difference in PE between *C. kuntzei* (mean = 393, 95% CI: 282–548) and *C. wilsonii* (mean = 458, 95% CI: 315–667; *z* = −0.894, *P* = 0.9481). By contrast, PE was significantly higher for *C. wilsonii* at mid‐ (mean = 704, 95% CI: 521–952) and high elevations (mean = 907, 95% CI: 692–1187) than for *C. kuntzei* at mid‐ (mean = 320, 95% CI: 233–441; *z* = −5.720, *P* < 0.0001) and high elevations (mean = 300, 95% CI: 214–422; *z* = −7.966, *P* < 0.0001). Within species, PE for *C. kuntzei* did not differ across elevations (low vs mid elevation: *z* = 0.928, *P* = 0.9394; mid vs high elevation: *z* = 0.296, *P* = 0.9997). By contrast, PE for *C. wilsonii* increased with elevation, with hummingbird effectiveness at high elevation being significantly higher than at both mid‐ (*z* = −1.266, *P* = 0.8039) and low elevation (*z* = −3.042, *P* = 0.0285). Additionally, PE for *C. wilsonii* at mid elevation (mean = 702, 95% CI: 519–950) was significantly higher than at low elevation (mean = 457, 95% CI: 314–665; *z* = −3.032, *P* = 0.0293).

## Discussion

Overall, our study shows that hummingbird pollination can represent a higher adaptive peak than bee pollination solely because of higher per‐visit pollen transfer efficiency, and that a subtle increase in hummingbird visitation, in this case, at higher elevation, can favor a pollination shift without any decline in bee visitation. Our results contrast with the longstanding hypothesis that shifts to hummingbird pollination in the American tropics are driven by low bee activity in montane environments (Cruden, 1972; Dellinger *et al*., 2021). Previous studies supporting that hypothesis did not experimentally translocate plants across elevations to directly assess floral adaptation and bee availability. They also focused on higher elevations than those occupied by *Costus* and did not examine plants specifically pollinated by euglossine bees. Consequently, their results may not extend to the lower end of the montane cloud forest or to certain bee taxa. Although declining bee availability at high elevation may explain some shifts to hummingbird pollination, we show that it is not a prerequisite, and the high efficiency of hummingbirds likely allows a broader range of conditions under which hummingbird pollination is favored.

Our results from both the naturally occurring plants and the translocations support the importance of hummingbird pollen transfer efficiency in creating a selective environment that would favor a shift to hummingbirds. Naturally occurring *C. wilsonii* received fewer pollinator visits per day than *C. kuntzeii* but had more pollen deposited on their stigmas. Moreover, in the translocated arrays at mid‐ and high‐elevation, similar visitation rates between bees and hummingbirds resulted in much higher overall effectiveness of hummingbirds. Our findings of higher hummingbird efficiency are consistent with studies in other systems, including North American *Penstemon* (Castellanos *et al*., 2003; Cardona *et al*., 2020), dry forest *Helicteres* and *Malvaviscus* (King *et al*., 2013), and Neotropical populations of *Digitalis purpurea* (Mackin *et al*., 2021), which show that hummingbirds deposit more pollen despite visiting less often than bees.

Because our design included only one site at each elevation, we cannot fully separate elevational effects from other site‐specific sources of variation. Accordingly, patterns described here as elevational may partly reflect unique characteristics of the sampled sites rather than elevation alone, although we sampled naturally occurring plants and array locations covering multiple hectares within each site to capture substantial within‐elevation variability. Moreover, the patterns found are unlikely to reflect unfamiliarity of pollinators with the experimental arrays. Multiple hermit hummingbird‐pollinated *Costus* species occur at low elevation, so local hermits should already be familiar with *Costus* flowers there. By contrast, no naturally occurring bee‐pollinated *Costus* occurs at the high‐elevation site, yet bee visitation to our arrays was just as high there as at low‐ and mid‐elevation sites. Thus, if unfamiliarity with the arrays reduced bee visitation at high elevation, visitation there should have been lower.

Our results help explain the numerous shifts from bee to hummingbird pollination that have occurred in the Neotropical *Costus* clade, including several shifts in lowland species. Even at the low elevation site where bee‐pollinated *C. kuntzei* is resident and hummingbird visitation to the arrays was low, the overall effectiveness of bees and hummingbirds was similar because of the high hummingbird efficiency. That similarity is consistent with a previous comparative study in *Costus* that found no consistent association between pollination syndrome and elevation (Vargas *et al*., 2020; Kay & Grossenbacher, 2022). Without bee pollination providing a clear, consistent advantage, plant populations at any elevation may be primed for a pollination shift with subtle increases in hummingbird visitation, even if hummingbird visits remain less frequent than bee visits.

Improved efficiency alone can favor a pollination shift even when ancestral pollinators remain abundant (Thomson & Thomson, 1992; Thomson, 2003; Ohashi *et al*., 2021). Kay & Anderson (2025) recently modeled an adaptive landscape in which equally abundant bees and hummingbirds visit a spectrum of floral phenotypes and showed that a very slight difference in the percent of removed pollen successfully transferred (from 2% by bees to 4% by hummingbirds) results in strong directional selection for an exclusively hummingbird‐pollinated flower, even through an intermediate phenotype that is less attractive to both pollinators. Based on anther size, *C. wilsonii* likely produces less pollen than *C. kuntzei*, so its higher per‐visit pollen receipt could easily represent a twofold increase in the percent of pollen successfully transferred.

Hummingbirds are likely more efficient at transferring pollen because they do not groom and consume pollen systematically the way bees do (Castellanos *et al*., 2003; Thomson, 2003). This difference may lead to higher pollen carryover by hummingbirds, facilitating broad pollen distribution across distant flowering individuals (Price & Waser, 1982; Castellanos *et al*., 2003; Thomson & Wilson, 2008; Krauss *et al*., 2017). By contrast, bees often deposit the most pollen on the first few flowers they visit while losing substantial amounts through grooming (Morris *et al*., 1994, 1995; Rademaker *et al*., 1997; Castellanos *et al*., 2003). High pollen transfer efficiency and pollen carryover can accrue fitness benefits through both female and male function, even without any pollen limitation (Thomson & Wilson, 2008; Krauss *et al*., 2017; Kay & Anderson, 2025). It can reduce geitonogamy or biparental inbreeding (Morris *et al*., 1994), resulting in higher quality offspring (Schemske, 1983; Schemske & Pautler, 1984; Schemske & Lande, 1985). The large pollen loads deposited by hummingbirds are likely to include genetically diverse mates and can promote pollen‐tube competition and mate choice (Willson & Burley, 1983; Walsh & Charlesworth, 1992). In *C. wilsonii*, a single hummingbird visit deposits sufficient pollen to fertilize all ovules many times over (mean ± SD = 30.5 ± 7.19 ovules per ovary), exceeding the critical pollen threshold required for successful pollen germination on *Costus* stigmas (Schemske & Fenster, 1983). By contrast, because *C. kuntzei* has more ovules per ovary (mean ± SD = 54.0 ± 12.41 ovules per ovary), a single visit only slightly exceeds the amount needed to fertilize all ovules. Differences in pollinator efficiency also may result in strong selection through male function, through effects on pollen export and siring success (Conner & Via, 1993; Brunet & Holmquist, 2009; Minnaar *et al*., 2019; Santana *et al*., 2025). Without the risk of grooming, plants may be able to make fewer pollen grains that can be removed in fewer overall visits for the same or greater siring success.

Hummingbirds also may generally fly longer distances than bees, promoting greater outcrossing and genetic cohesion, although this is unlikely to be a major factor driving hummingbird pollination in *Costus*. Gamba & Muchhala (2023) showed that hummingbird‐pollinated plants had less population differentiation than bee‐pollinated plants, except for the one euglossine‐pollinated species studied. Both euglossine bees and hermit hummingbirds are known to forage over long distances among spatially isolated plants (Janzen, 1971; Gill, 1988; Torres‐Vanegas *et al*., 2019). Nevertheless, foraging distances and pollen carryover in euglossine bees deserve further study (Opedal *et al*., 2017).

Although we did not find a decline in bee visitation with elevation, we did find an increase in hummingbird visitation. Some hummingbird taxa are more abundant at higher elevations compared to lowlands (Stiles, 1981, 2008), where they are physiologically adapted to cold, hypoxic conditions (Stiles, 1981; Altshuler *et al*., 2004a, 2004b; Projecto‐Garcia *et al*., 2013). Their high metabolic capacity allows foraging under low temperatures and frequent precipitation (Suarez & Gass, 2002; Altshuler *et al*., 2004a, 2004b). In addition, the prevalence of nectar‐rich flowers in montane habitats may reinforce hummingbird diversity and abundance through reciprocal plant–pollinator coevolution (Feinsinger, 1983; McGuire *et al*., 2014; Rico‐Guevara *et al*., 2021). However, these studies focused on nonhermit hummingbirds rather than the Phaethornithinae, a predominantly low‐elevation clade in which no morphological or aerodynamic traits show significant variation with elevation, in contrast to the significant elevational shifts in wing size, and foot and tarsus dimensions documented in many nonhermits that indicate adaptation to montane conditions (Stiles, 2004, 2008). Thus, our finding of increasing hummingbird visitation with elevation remains unexplained and may have more to do with the availability of other food resources, as has been found across seasonal changes in lowland habitats (Stiles, 1978; Grove, 1985).

In conclusion, we provide empirical evidence that a pollination shift can arise through adaptation to more efficient pollinators, rather than through reduced visitation of ancestral pollinators. Our field observations and reciprocal translocations do not support adaptation to hummingbirds being driven by declining bee visitation with elevation, as has long been proposed for the American tropics. We highlight the importance of looking beyond pollinator visitation rates and incorporating pollen transfer efficiency to better understand major evolutionary transitions in floral phenotypes.

## Competing interests

None declared.

## Author contributions

PJ and KMK contributed to conceptualization. PJ, KG, EH, CG, AD, DT, AL and KMK were involved in the investigation of the study. PJ contributed to data curation. PJ contributed to formal analysis. PJ and KMK contributed to funding acquisition. PJ contributed to visualization. PJ and KMK contributed to writing – original draft. PJ, KG, EH, CG, AD, DT, AL and KMK contributed to writing – review and editing.

## Disclaimer

The New Phytologist Foundation remains neutral with regard to jurisdictional claims in maps and in any institutional affiliations.

## Supporting information


**Table S1** Floral trait comparisons.
**Table S2** Overall pollinator visitation sampling details.
**Table S3** Pollinator visitation sampling details by year.
**Table S4** Pollinator per‐visit efficiency sampling details.
**Table S5** Overall sampling details of the reciprocal translocation of floral arrays.
**Table S6** Sampling details of the reciprocal translocation of floral arrays by year.
**Table S7** Model output for overall pollinator visitation rate across species.
**Table S8** Model output for overall pollinator per‐visit efficiency across species.
**Table S9** Model output for overall pollinator effectiveness across species.
**Table S10** Model output for pollinator visitation rate across species and elevations.
**Table S11** Model output for pollinator per‐visit efficiency across species and elevations.
**Table S12** Model output for pollinator effectiveness across species and elevations.
**Table S13** Model output for visitation rate in translocations across species and sites.
**Table S14** Model output for effectiveness in translocations across species and sites.
**Methods S1** Species occurrences and elevational distribution.

**Video S1** Video compilation of pollinator observations.
Please note: Wiley is not responsible for the content or functionality of any Supporting Information supplied by the authors. Any queries (other than missing material) should be directed to the *New Phytologist* Central Office.

## Data Availability

All data and analysis scripts are available in the Dryad Digital Repository at https://doi.org/10.5061/dryad.931zcrk0r.
