# Supplementary material for: Pollinator efficiency, rather than bee decline, explains a shift to hummingbird pollination in tropical montane forests
Source: New Phytol. 2026 Jun 4;251(4):2237–47. doi: 10.1111/nph.71291 (PMC13373865; doi:10.1111/nph.71291)
Supplement: Supplementary file 1 — Table S1 Floral trait comparisons. Table S2 Overall pollinator visitation sampling details. Table S3 Pollinator visitation sampling details by year. Table S4 Pollinator per‐visit efficiency sampling details. Table S5 Overall sampling details of the reciprocal translocation of floral arrays. Table S6 Sampling details of the reciprocal translocation of floral arrays by year. Table S7 Model output for overall pollinator visitation rate across species. Table S8 Model output for overall pollinator per‐visit efficiency across species. Table S9 Model output for overall pollinator effectiveness across species. Table S10 Model output for pollinator visitation rate across species and elevations. Table S11 Model output for pollinator per‐visit efficiency across species and elevations. Table S12 Model output for pollinator effectiveness across species and elevations. Table S13 Model output for visitation rate in translocations across species and sites. Table S14 Model output for effectiveness in translocations across species and sites. Methods S1 Species occurrences and elevational distribution. [file NPH-251-2237-s002.pdf]

New Phytologist

New Phytologist **Supporting Information**

Article title: Pollinator efficiency, rather than bee decline, explains a shift to hummingbird pollination in tropical montane forests

Authors: Pedro Juárez, Kathryn Gerhardt, Eden Hughes, Cecilia Girvin, Anise Dellith-Moser, Dennis Tenorio, Annya Livak, & Kathleen M. Kay

Article acceptance date: 01 May 2026

The following Supporting Information is available for this article:

Table S1 Floral trait comparisons.

Table S2 Overall pollinator visitation sampling details.

Table S3 Pollinator visitation sampling details by year.

Table S4 Pollinator per-visit efficiency sampling details.

Table S5 Overall sampling details of the reciprocal translocation of floral arrays.

Table S6 Sampling details of the reciprocal translocation of floral arrays by year.

Table S7 Model output for overall pollinator visitation rate across species.

Table S8 Model output for overall pollinator per-visit efficiency across species.

Table S9 Model output for overall pollinator effectiveness across species.

Table S10 Model output for pollinator visitation rate across species and elevations.

Table S11 Model output for pollinator per-visit efficiency across species and elevations.

Table S12 Model output for pollinator effectiveness across species and elevations.

Table S13 Model output for visitation rate in translocations across species and sites.

Table S14 Model output for effectiveness in translocations across species and sites.

Methods S1 Species occurrences and elevational distribution.

Video/Movie S1 Video compilation of pollinator observations.

Table S1 Floral traits mean comparisons between Costus kuntzei and C. wilsonii, with multiple two-tailed t tests and adjusting p values using Bonferroni correction. Values are reported as: mean ± SD (N), where N is the total number of flowers measured. Abbreviations: t = t statistic, df = degrees of freedom, and P = p value.

Floral traits	*C. kuntzei*	*C. wilsonii*	t	df	P
Petal length (cm)	3.78 ± 0.28 (27)	2.72 ± 0.28 (36)	14.81	56.77	0.000
Labellum length (cm)	3.48 ± 0.77 (33)	1.35 ± 0.77 (41)	11.76	68.58	0.000
Labellum width (cm)	1.39 ± 0.24 (27)	0.72 ± 0.14 (37)	12.89	37.9	0.000
Striation area (cm^2)	3.18 ± 1.00 (39)	0.25 ± 0.61 (41)	15.69	62.12	0.000
Tube length (cm)	1.42 ± 0.27 (33)	0.89 ± 0.23 (41)	9.09	63.37	0.000
Tube diameter (cm)	0.40 ± 0.20 (27)	0.29 ± 0.04 (37)	2.63	27.66	0.221
Curvature (cm)	2.82 ± 0.10 (33)	2.49 ± 0.19 (41)	9.34	62.87	0.000
Filament length (cm)	4.67 ± 0.45 (33)	4.31 ± 0.30 (41)	4.04	53.27	0.003
Anther length (cm)	0.68 ± 0.13 (33)	0.54 ± 0.08 (41)	5.49	49.75	0.000
Style length (cm)	4.28 ± 0.48 (33)	3.54 ± 0.25 (41)	8.03	45.22	0.000
Gullet length (cm)	1.43 ± 0.22 (33)	1.28 ± 0.18 (41)	3.14	63.26	0.042
Gullet width (cm)	0.68 ± 0.14 (33)	0.50 ± 0.07 (41)	6.66	44.4	0.000
Ovary area (cm^2)	0.44 ± 0.11 (17)	0.26 ± 0.13 (16)	4.14	29.37	0.004
Ovule number	54.00 ± 12.41 (17)	30.50 ± 7.19 (16)	6.7	25.93	0.000
Nectar volume (mm^3)	11.89 ± 11.38 (19)	34.42 ± 9.32 (13)	-6.13	28.91	0.000
Sugar concentration (°Bx)	26.18 ± 7.02 (19)	27.92 ± 2.89 (13)	-0.97	25.67	1.000

Table S2 Pollinator visitation sampling details for Costus kuntzei and C. wilsonii. N is the number of individuals.

Species	Site	Number of plants	Visits per day mean ±SD (N)
	Low	16	3.64 ± 5.59 (33)
	High	19	10.26 ± 6.33 (34)
C. kuntzei	Overall	34	7.00 ± 6.80 (67)
	Low	26	1.88 ± 1.63 (34)
	High	19	2.05 ± 1.70 (42)
C. wilsonii	Overall	40	1.97 ± 1.66 (76)

Table S3 Pollinator visitation sampling details by year for Costus kuntzei and C. wilsonii. N is the number of individuals.

Species	Site	Year	Number of plants	Visits per day mean ± SD (N)
C. kuntzei	Low	2018	4	4.50 ± 8.19 (14)
		2024	12	3.00 ± 2.47 (19)
	Mid	2018	3	4.00 ± 3.00 (5)
		2019	1	10.25 ± 9.00 (4)
		2024	16	11.52 ± 5.80 (25)
	Overall	2018	6	4.37 ± 7.10 (19)
		2019	1	10.25 ± 9.00 (4)
		2024	28	7.84 ± 6.29 (44)
C. wilsonii	Mid	2018	3	0.33 ± 0.58 (3)
		2021	9	1.60 ± 1.51 (10)
		2024	14	2.24 ± 1.67 (21)
	High	2018	5	1.67 ± 2.73 (6)
		2024	14	2.11 ± 1.51 (36)
	Overall	2018	5	1.22 ± 2.28 (9)
		2021	9	1.60 ± 1.51 (10)
		2024	28	2.16 ± 1.56 (57)

Table S4 Pollinator per-visit efficiency (stigma pollen load per visit) sampling details for Costus kuntzei and C. wilsonii. N is the number of stigmas analyzed.

Species	Site	Year	Visits per day mean ±SD		Per-visit pollen load mean ±SE (N)		
C. kuntzei	Low	2024	3.31	±2.5	85.1	±22.4	(16)
	Mid	2024	11.5	±5.69	67.4	±16.1	(26)
	Overall		8.36	±6.17	75.2	±16.3	(42)
C. wilsonii	Mid	2024	2.69	±1.58	160	±0 38	(16)
	High	2024	2.14	±1.48	118.2	±26.2	(35)
	Overall		2.31	±1.52	134	±26.7	(51)

Table S5 Sampling details of the reciprocal translocation of floral arrays for Costus kuntzei and C. wilsonii. N is the total number of visits at each array site.

Site	Number of array locations	*C. kuntzei* Bee visits per day mean ±SD (N)	*C. wilsonii* Hummingbird visits per day mean ±SD (N)
High	7	7.18 ±7.88 (244)	6.44 ±5.77 (219)
Mid	7	4.38 ±3.8 (162)	3.59 ±3.46 (133)
Low	7	8.52 ±10.7 (264)	4.68 ±3.45 (145)

Table S6 Sampling details of the reciprocal translocation of floral arrays by year for Costus kuntzei and C. wilsonii. N is the total number of visits at each array site.

Site	Year	Number of array locations	*C. kuntzei* Bee visits per day mean ±SD (N)	*C. wilsonii* Hummingbird visits per day mean ±SD (N)
	2022	2	1.92 ± 2.07 (23)	6.67 ± 4.70 (80)
High	2023	5	7.68 ± 7.24 (169)	5.23 ± 4.93 (115)
	2021	2	4.40 ± 4.45 (44)	3.40 ± 2.67 (34)
	2022	1	6.40 ± 5.46 (32)	7.20 ± 2.05 (36)
Mid	2023	4	3.77 ± 2.93 (83)	2.41 ± 2.89 (53)
	2022	6	9.18 ± 11.02 (257)	3.04 ± 2.20 (85)
Low	2023	1	1.33 ± 0.58 (4)	0.00 ± 0.00 (0)

Table S7 Model output for overall comparisons of pollinator visitation rate between Costus kuntzei and C. wilsonii in the wild dataset. Plant identity and year were included as random effects, and observation duration was included as an offset.

Model	Output				
Fixed Effects:		Estimate	Std. Error	z-value	Pr(>|z|)
visits ~ species	(Intercept)	1.045	0.186	5.62	1.86E-08
	Species(wilsonii)	-1.252	0.165	-7.588	3.25E-14

Table S8 Model output for overall comparisons of pollinator per-visit efficiency between Costus kuntzei and C. wilsonii. Stigma identity was included as a random effect, and observation duration was included as an offset.

Model		Output			
Fixed Effects:		Estimate	Std. Error	z-value	Pr(>|z|)
no.pollen grains ~ species	(Intercept)	3.544	0.217	16.320	0.000
	Species(wilsonii)	0.578	0.181	3.193	0.001

Table S9 Model output for overall comparisons of pollinator effectiveness between Costus kuntzei and C. wilsonii in the wild dataset. Plant identity was included as a random effect, and observation duration was included as an offset.

Model		Output			
Fixed Effects:		Estimate	Std. Error	z-value	Pr(>|z|)
pollinator_effectiveness ~ species	(Intercept)	4.783	0.104	45.902	0
	Species(wilsonii)	0.384	0.087	4.397	1.10E-05

Table S10 Model output for comparisons of pollinator visitation rate between Costus kuntzei and C. wilsonii across elevations in the wild dataset. Plant identity and year were included as random effects, and observation duration was included as an offset.

Model		Output			
Fixed Effects:		Estimate	Std. Error	z-value	Pr(>|z|)
visits ~ species*elevation	(Intercept)	0.442	0.149	2.965	0.003
	Species(wilsonii)	-0.509	0.228	-2.231	0.026
	Elevation(higher)	1.072	0.196	5.465	4.64E-08
	Species(wilsonii):elevation (higher)	-1.100	0.301	-3.650	2.63E-04

Table S11 Model output for comparisons of pollinator per-visit efficiency between Costus kuntzei and C. wilsonii across elevations. Stigma identity was included as a random effect, and observation duration was included as an offset.

**Model**		**Output**			
Fixed Effects:		Estimate	Std. Error	z-value	Pr(>|z|)
no.pollen grains ~ species*elevation	(Intercept)	3.668	0.264	13.917	0
	Species(wilsonii)	0.631	0.287	2.195	0.028
	elevation(higher)	-0.233	0.267	-0.872	0.383
	Species(wilsonii):elevation(higher)	-0.069	0.359	-0.193	0.847

Table S12 Model output for comparisons of pollinator effectiveness between Costus kuntzei and C. wilsonii across elevations in the wild dataset. Plant identity was included as a random effect, and observation duration was included as an offset.

**Model**		**Output**			
Fixed Effects:		Estimate	Std. Error	z-value	Pr(>|z|)
pollinator_effectiveness ~ species*elevation	(Intercept)	4.601	0.131	35.203	0.000
	Species(wilsonii)	0.627	0.136	4.620	0.000
	elevation(higher)	0.351	0.137	2.556	0.011
	Species(wilsonii):elevation (higher)	-0.460	0.174	-2.642	0.008

Table S13 Model output for comparisons of pollinator visitation rate in reciprocal translocation floral arrays of Costus kuntzei and C. wilsonii across sites. Array identity and year were included as random effects, and array observation duration was included as an offset. LC is Las Cruces Biological Station, and LA is Las Alturas Biological Station.

**Model**		**Output**			
Fixed Effects:		Estimate	Std. Error	z-value	Pr(>|z|)
visits ~ species * site	(Intercept)	0.952	0.299	3.185	0.001
	Species(wilsonii)	-1.409	0.225	-6.267	0
	Site(LC)	-0.305	0.398	-0.767	0.443
	Site(LA)	-0.069	0.398	-0.173	0.862
	Species(wilsonii):site(LC)	1.185	0.322	3.678	0
	Species(wilsonii):site(LA)	1.452	0.309	4.695	0
Zero inflation:		Estimate	Std. Error	z-value	Pr(>|z|)
~ species * site	(Intercept)	-0.899	0.427	-2.105	0.035
	Species(wilsonii)	-18.851	4906.423	-0.004	0.997
	Site(LC)	-1.253	0.823	-1.523	0.128
	Site(LA)	-0.681	0.671	-1.014	0.31
	Species(wilsonii):site(LC)	19.394	4906.423	0.004	0.997
	Species(wilsonii):site(LA)	18.886	4906.423	0.004	0.997

Table S14 Model output for comparisons of pollinator effectiveness in reciprocal translocation floral arrays of Costus kuntzei and C. wilsonii across sites. Array identity was included as a random effect, and array observation duration was included as an offset. LC is Las Cruces Biological Station, and LA is Las Alturas Biological Station.

**Model**		**Output**			
Fixed Effects:		Estimate	Std. Error	z-value	Pr(>|z|)
pollinator_effectiveness ~ species * site	(Intercept)	5.026	0.169	29.653	0.000
	Species(wilsonii)	0.154	0.170	0.903	0.366
	Site(LC)	-0.200	0.221	-0.905	0.365
	Site(LA)	-0.274	0.225	-1.219	0.223
	Species(wilsonii):site(LC)	0.634	0.222	2.857	0.004
	Species(wilsonii):site(LA)	0.961	0.225	4.272	0.000

Methods S1 Species occurrences and elevational distribution

Occurrence data for both species in Fig. 1 were obtained from herbarium databases (*Herbario Nacional de Costa Rica*, https://biodiversidad.museocostarica.go.cr, *The Missouri Botanical Garden Herbarium*, https://tropicos.org). To ensure accuracy and reduce potential misidentifications, only occurrences identified by experts in the genus *Costus* in Costa Rica (i.e., B. Hammel, P. Juárez, K. M. Kay, P. Maas, and D. Skinner) were considered. We bounded the occurrence data within coordinates 08° to 11° latitude and -86° to -82° longitude, covering the region from the northern terrestrial border of Costa Rica to northwestern Panama. This resulted in 204 specimens for *C. kuntzei* and 63 specimens for *C. wilsonii*. Although the actual distribution of *C. kuntzei* extends beyond these boundaries, we focused on comparing elevation among co-occurring populations within the same geographical area. We extracted geographic coordinates from the occurrence data to obtain elevation data using the get_elev_point function in the *elevatr* R package (Hollister *et al.*, 2023). We then performed a Wilcoxon rank-sum test to compare median elevation between species.

Video/Movie S1 Compilation of pollinator observation videos across *Costus* species and elevations sites.

References

Hollister JW, Robitaille AL, Beck MW, Shah T, Nowosad J. **2023**. jhollist/elevatr: CRAN Release v0. 99.0. *Zenodo*.
